# Frequency of Intestinal Parasitic Infection and Associated Factors Among HIV Patients on Antiretroviral Therapy at Debre Tabor Comprehensive Specialized Hospital, Northwest Ethiopia: A 5‐Year Retrospective Study

**DOI:** 10.1155/jotm/1173013

**Published:** 2026-06-22

**Authors:** Dessie Tegegne, Mulu Kebede, Getaneh Tegegne, Yikeber Abateneh, Teklehaimanot Kiros, Mulat Erkihun, Andargachew Almaw, Ayenew Assefa, Ayenew Berhan, Alemie Fentie, Tegenaw Tiruneh, Birhanemaskal Malkamu, Mahider Shimelis Feyisa, Biruk Legese, Sisay Getu, Mitikie Wondimagegn Meberat, Birhanu Getie, Shewaneh Damtie, Bekele Sharew, Getu Abeje

**Affiliations:** ^1^ Debre Tabor University, Debre Tabor, Amhara, Ethiopia, dtu.edu.et; ^2^ Injibara University, Injibara, Amhara, Ethiopia, inu.edu.et

**Keywords:** ART, DTCSH, Ethiopia, HIV, intestinal parasites

## Abstract

**Background:**

Intestinal parasite infections (IPIs) are significant public health concerns, particularly for those with HIV/AIDS. Low hygiene standards, tainted water, and inadequate sanitation all contribute to Ethiopia’s high frequency of IPIs. HIV patients are still susceptible to several parasitic illnesses even after antiretroviral medication (ART) and deworming efforts have been implemented. The data in the study area are restricted, despite the fact that few studies describe IPIs among HIV patients. Furthermore, by taking sociodemographic and clinical aspects into account, this study produced thorough 5‐year data. In order to fill in the gaps in local epidemiological data and guide focused treatments, this study evaluated the frequency of IPIs and the factors that are linked to them among HIV patients receiving ART.

**Objective:**

To assess the frequency of IPIs and related variables in HIV/AIDS patients receiving ART at Debre Tabor Comprehensive Specialized Hospital (DTCSH), Ethiopia.

**Methods:**

A 5‐year retrospective hospital‐based study was conducted using medical records of 600 HIV patients on ART at DTCSH. Included were data registered between May 2020 and May 2025. Data on sociodemographic, CD_4_ counts, ART duration, and IPIs were extracted from patient cards using a convenient sampling technique. SPSS Version 26 was utilized for data entry and analysis. Descriptive statistics along with bivariate and multivariable analyses were conducted. All variables in the multivariable logistic regression were deemed statistically significant if their *p* value was less than 0.05 at a 95% confidence interval. Text, tables, and graphs were used to display the data.

**Results:**

Out of 600 records, 33.5% of people have IPIs. *Entamoeba histolytica* (59.7%) and *Giardia lamblia* (33.3%) were the most common parasites found. Individuals with age over 60 years and those living in rural areas had considerably higher IPIs (*p* < 0.05). However, when comparing patient records examined in the first year to those in the fifth, the frequency of IPIs declined. Furthermore, those with low CD_4_ counts (less than 200 cells/μL), advanced WHO clinical stages (3‐4), and shorter ART durations (less than 5 years) had significantly higher likelihood of IPIs (*p* < 0.05).

**Conclusion:**

Among HIV patients receiving ART, IPIs remain prevalent. Important determinants of IPIs include age, place of residency, CD_4_ count, WHO stages, and adherence to ART. Long‐term ART adherence has shown protective advantages, underscoring its importance in reducing the burden of IPIs. Enhancing the management of parasitic infections and HIV requires integrated strategies that include improved sanitation, frequent testing, and health education. Future large‐scale research is also necessary.

## 1. Introduction

One of the major causes of morbidity and mortality worldwide, especially for HIV/AIDS patients, is intestinal parasite infections (IPIs) [1–3]. Because of their compromised immune systems, these people are particularly vulnerable to a variety of illnesses, including intestinal parasites (IPs) [3, 4]. The global reports state that there were 863,840 deaths, 36.85 million HIV/AIDS cases, [5], and incidence of 1.65 million in 2021 with an increased burden in sub‐Saharan Africa [6]. Due to its high prevalence and huge population impact, HIV/AIDS continues to be a major public health concern in Ethiopia [7]. According to one report, there are 617,921 HIV/AIDS‐positive individuals in Ethiopia, including 235,550 men and 382,371 women of all ages [8].

Over one billion people worldwide suffer from IP infestations [9]. IPs are also common problems in developing countries, especially in Africa, which are mostly linked with low socioeconomic status, food contamination, environmental and personal hygiene, lack of awareness on health seeking behaviors, and limited access to potable water [10, 11].

The co‐occurrence of HIV/AIDS and lower economy level makes this dual HIV/AID‐IPIs burden particularly dangerous in underdeveloped countries such as Ethiopia [12]. One study reported in 2024 that the overall IPIs was higher (19.3%) among all participants of HIV/AIDS patients compared to those with a CD_4_+ T‐cell count below 200 cells/μL [13]. Coinfections with IPs remain a major societal burden despite advancements in HIV/AIDS prevention and treatment [14]. It has been demonstrated that IPIs worsen progression to the advanced stages of AIDS, making individuals more vulnerable to opportunistic infections and lowering their life expectancy rates [15–17].

Although earlier cross‐sectional studies and systematic reviews indicated that the frequency of IPIs among individuals living with HIV/AIDS ranged from 19.3% to 42.24% [4, 13, 18, 19], there is a dearth of a current, thorough retrospective investigation that addresses these issues in developing nations such as Ethiopia. Additionally, the current study took into account variables that may be connected to susceptibility to infectious agents, such as WHO stages, ART adherence, and CD_4_ count level. Therefore, this study used a 5‐year record data set to examine the frequency of IPs and related variables among HIV/AIDS patients on ART. By filling these gaps, the results offered crucial epidemiological information and contributed to the body of knowledge by emphasizing the continued difficulty of IPIs and offering integrated parasite screening as part of HIV patient care programs.

## 2. Objectives

### 2.1. General Objective

To assess the frequency of IPs and associated factors among HIV/AIDS patients on ART at DTCSH, Northwest Ethiopia, based on records filed between May 2020 and May 2025.

### 2.2. Specific Objectives

To identify the factors that could be associated with IPIs among HIV patients on ART at DTCSH, Northwest Ethiopia based on records filed between May 2020 and May 2025.

## 3. Methods and Materials

### 3.1. Study Area

This study was carried out at DTCSH, Northwest Ethiopia. Located in Debre Tabor, the hospital is roughly 589 km from Addis Ababa and 104 km from Bahir Dar. For more than 5 million residents of South Gondar and the surrounding areas, the hospital offers a broad range of medical services, including emergency and trauma care, outpatient and inpatient care, surgical care, maternity and child health care, and mental health services. As a specialty hospital, it offers ART and acts as a referral center for a number of illnesses, including HIV/AIDS.

### 3.2. Study Design and Period

The medical records of HIV patients receiving ART from May 2020 to May 2025 were examined as part of an institution‐based retrospective study design that ran from May 30 to June 30, 2025.

### 3.3. Population

The source population consisted of all HIV/AIDS patients who attended the ART clinic, while the study population consisted of all HIV/AIDS patients who received ART in the hospital between May 2020 and May 2025.

### 3.4. Eligibility Criteria

Medical records with comprehensive information on IPIs and related variables, as well as patients who have been on ART for at least 6 months, were included, while incomplete or missing information from patient cards or medical records was excluded.

### 3.5. Sampling Technique

To choose and obtain the necessary sample size, a convenient sampling procedure was employed.

### 3.6. Variables

#### 3.6.1. The Dependent Variable

The frequency of IPIs was used as the dependent variable in this study.

### 3.7. The Independent Variables

The independent variables include clinical variables (duration on ART, CD_4_ count, and WHO clinical stage) and sociodemographic factors (age, sex, occupation, and place of residence).

### 3.8. Data Collection

Lab reports of IPs and data were collected using a semistructured checklist. Clinical characteristics such as CD_4_ counts (defined as < 200, 200–500, and > 500 cells/μL), WHO clinical staging [1–4], and duration on ART were extracted. Additionally, sociodemographic data such as age, sex, occupation, and address included.

### 3.9. Data Processing and Analysis

Data were cleaned, entered, and analyzed using SPSS Version 26. Descriptive statistics were used to summarize sociodemographic and clinical characteristics, while the frequency of IP was calculated as a proportion of infected patients relative to the total sample size. Binary and multivariable logistic regression analyses were used to assess the association between dependent and independent variables. The multivariable logistic regression model was fitted to variables with a *p* value < 0.25 in the binary logistic regression analysis. In the multivariable logistic regression, variables with an adjusted odds ratio (AOR) at the 95% CI and a *p* value < 0.05 was deemed statistically significant.

### 3.10. Data Quality Control

Cross‐checking with the original hospital records was done to address any inconsistencies or missing data in order to guarantee the accuracy and dependability of the data gathered. To ensure consistency in the recording of variables such as CD_4_ counts, ART duration, and parasite diagnostic results, a semistructured data extraction checklist was employed. To reduce errors, double data entry was used. Inconsistencies were then found and fixed using validation. To ensure data integrity, the lead investigator audited the dataset on a regular basis.

## 4. Results

### 4.1. Social Demographics and Clinical Information

Most of the 600 patient records were from women (64.8%) and individuals between the ages of 45 and 59 (30.3%). Similarly, the majority of survey participants were merchants (40.2%) and urban dwellers (94.8%). Clinically, a CD_4_ count of more than 500 cells/mm3 of blood was present in almost half (49.2%) of the participants. Additionally, the majority of individuals (72.7%) were in the first level of WHO staging (35.2%) and had been on ART for 10–15 years (see Table 1).

**TABLE 1:** Frequency distribution of sociodemographic and clinical data.

Variables	Categories	Frequencies (%)
Sex	Male	211 (35.2)
	Female	389 (64.8)

Age	< 15	31 (5.2)
	16–29	72 (12)
	30–44	158 (26.3)
	45–59	182 (30.3)
	≥ 60	157 (26.2)

Address	Urban	569 (94.8)
	Rural	31 (5.2)

Occupation	Farmer	90 (15)
	Merchant	241 (40.2)
	House wife	121 (20.2)
	Employed	148 (24.7)

Patient assessment year	In the 1^st^ year	171 (28.5)
	In the 2^nd^ year	119 (19.83)
	In the 3^rd^ year	104 (17.33)
	In the 4^th^ year	112 (18.67)
	In the 5^th^ year	94 (15.67)

CD_4_ count	< 200	51 (8.5)
	200–500	254 (42.3)
	> 500	295 (49.2)

WHO stages [1–4]	T1 and T2	480 (80%)
	T3 and T4	120 (20%)

Duration on ART in years	< 5	41 (6.8)
	6–10	200 (33.3)
	10–15	211 (35.2)
	16–20	101 (16.8)
	> 20	47 (7.8)

### 4.2. Frequency of IPs

The frequency of IPIs was 33.5%. Females exhibited a higher IPI (33.67%). Out of these positives, *Entamoeba histolytica* (59.7%) and *Giardia lamblia* (33.3%) were predominantly identified. Interestingly, the IPI prevalence was higher in the age groups of over 60 years (35%). Clinically, the parasite positivity rate was relatively increased for people in WHO Stages T3 and T4 at 36.9% and 36.4%, respectively. Most of the study participants with a CD_4_ count < 500 had an increased IPI (35.4%) (Table 2).

**TABLE 2:** Factors linked with IPIs among HIV AIDS patients on ART at DTCSH, Northwest Ethiopia, 2025.

Variables		Frequency (%)	IP negative (%)	IP positive (%)	COR (95% CI)	*p* value	AOR (95% CI)	*p* value
Sex	Male	211 (35.2)	141 (66.8)	70 (33.2)	1		1	
	Female	389 (64.8)	258 (66.3)	131 (33.7)	1.37 (0.9–2.05)	0.15	1.42 (0.94–2.15	0.09

Age in years	< 15	31 (5.2)	21 (67.7)	10 (32.3)	1		1	
	16–29	72 (12)	48 (66.7)	24 (33.3)	0.41 (0.15–1.2)	0.34	0.45 (0.16–1.3)	0.12
	30–44	158 (26.3)	107 (67.7)	51 (32.3)	0.09 (0.03–0.3)	0.02	0.1 (0.03–1.3)	0.1
	45–59	182 (30.3)	121 (66.5)	61 (33.5)	1.58 (0.8–3.5)	0.23	1.5 (0.9–3.05)	0.4
	≥ 60	157 (26.2)	102 (65%)	55 (35%)	1.8 (2.3–4.5)	0.03	0.72 (0.24–2.2)	0.2

Residence	Urban	569 (94.8)	378 (66.4)	191 (33.6)	1		1	
	Rural	31 (5.2)	21 (67.7)	10 (32.3)	1.08 (1.02–6.5)	0.012	3 (1.08–5.05)	^∗^0.03

Occupation	Farmer	90 (15)	62 (69)	28 (31)	1		1	
	Merchant	121 (40.2)	83 (68.6)	38 (31.4)	1.25 (0.8–2.1)	0.13	1.30 (0.77–2.2)	0.33
	House wife	241 (20.2)	163 (67.6)	78 (32.4)	0.33 (0.2–0.6)	0.01	0.35 (0.2–1.7)	0.1
	Employed	148 (24.7)	91 (61.5)	57 (38.5)	0.62 (0.34–1.2)	0.15	0.65 (0.35–1.2)	0.17

Parasite identification year	1^st^ year	171 (28.5)	94 (55)	77 (45)	1		1	
	2^nd^ year	119 (19.83)	72 (60.5)	47 (39.5)	1.01 (0.5–1.9)	0.9	1.5 (0.2–3.6)	0.85
	3^rd^ year	104 (17.33)	75 (72)	29 (28)	0.8 (0.4–1.5)	0.47	0.6 (0.15–6.4)	0.7
	4^th^ year	112 (18.67)	86 (76.8)	26 (23.2)	0.47 (0.3–0.9)	0.014	0.34 (0.16–0.8)	^∗^0.02
	5^th^ year	94 (15.67)	72 (76.6)	22 (23.4)	0.37 (0.21–0.7)	0.01	0.44 (0.21–0.9)	^∗^0.01

CD_4_ count (cells/μL)	< 200	51 (8.5)	42 (82.4)	9 (17.6)	4.9 (2.45–9.8)	0.02	5.15 (2.6–10.4)	^∗^0.001
	200–500	254 (42.3)	155 (61)	99 (39)	1.98 (1.05–3.7)	0.04	2.05 (1.08–3.9)	^∗^0.03
	> 500	295 (49.2)	244 (96)	10 (4)	1		1	

WHO staging	T3 and T4	120 (20%)	86 (72.7)	34 (28.3)	8.1 (7.5–29.2)	0.002	9.2 (8.3–31.8)	^∗^0.001
	T1 and T2	480 (80)	464 (96.7)	16 (3.3)	1		1	

Duration on ART	0–5	41 (6.8)	32 (78)	9 (23)	11 (5.3–22.6)	0.001	11.5 (5.5–24.0)	^∗^0.001
	6–10	200 (33.4)	190 (95)	10 (5)	2.50 (1.2–5.2)	0.03	2.6 (1.25–5.4)	^∗^0.01
	> 10	359 (59.8)	342 (95)	17 (5)	1		1	

Abbreviations: AOR, adjusted odd ratio; CI, confidence interval; COR, crude odd ratio.

^∗^Statistically significant.

### 4.3. IPIs and Factors Among HIV/AIDS Patients on ART

The prevalence of IPs were almost five times more common in individuals with CD_4_ levels under 200 cells/μL than in those with counts above 500 cells/μL (AOR: 5.15, 95% CI: 2.55–10.40, *p* = 0.001). Additionally, individuals with advanced WHO clinical stages (3 and 4) had nine times the odds of IPIs (AOR: 9.2 (95% CI: 8.3–31.8, *p* < 0.001). Patients on ART for ≤ 5 years had also 11.5 times greater chances of IPIs than those on ART for > 10 years (AOR: 11.50, 95% CI: 5.50–24.00, *p* < 0.001). The likelihood of IP infection was higher among participants who lived in rural areas than those in urban residents (AOR: 3, 95% CI: 1.08–5.05, *p* = 0.03) (Table 2).

## 5. Discussion

The current study found that among HIV patients on ART at DTCSH, the frequency of IPIs was high, at 33.5% (95% CI: 29.3%–37.1%), which was in line with previous findings from Bahir Dar (30.6%) [20], Gondar (29.1%) [21], and Hosanna Health Center, Ethiopia 31.5% [1]. This significantly high frequency of IPIs other than opportunistic parasites demonstrated the continued public health importance of parasites among HIV/AIDS patients after starting ART. Nevertheless, our findings were comparatively higher than those reported by Debre Tabor (25.3%) [18], as discussed by Aksum (24.6%) [22] and Butajira (26.6%) [23]. The observed differences may be ascribed to changes in water quality, sanitation, and the efficacy of regional deworming programs over time. The frequency IPs in the present study was lower than that reported from Ethiopia 39.25%–40.24% [4, 19], Burkina Faso (65.3%) [24], Cameroon (82.3%) [25], and Mexico (69%) [26]. These discrepancies underscore the crucial role of ART compliance, socioeconomic circumstances, and restricted access to medical treatment, especially in environments with minimal resources. It could also be because people have become more conscious over time.

A strong relationship between CD_4_ counts and IPIs was found in our study; patients with CD_4_ counts below 500 cells/μL had a higher infection incidence (35.4%) than patients with high‐level CD_4_ counts (> 500 cells/μL). This emphasizes even more how crucial cellular immunity is to controlling these parasite diseases. This study demonstrated the crucial role that severe immunosuppression and later stages of AIDS play in making patients more susceptible to both opportunistic and nonopportunistic parasites. This finding is consistent with two studies carried out in Ethiopia from Butajira [23] and Arba Minch [27]. Precisely, our investigation found that among all IP positives, protozoan parasites, notably *Entamoeba histolytica* (59.7%) and *Giardia lamblia* (33.3%), were more common than helminths (*A. lumbricoides*, 7%). Accordingly, prior research had documented a preponderance of protozoan parasites, particularly *E. histolytica* and *G. lamblia*, among HIV/AIDS patients [22, 23]. On the other hand, a previous finding from Hossana Health Center Ethiopia had shown a higher frequency of helminths than protozoan parasites [1]. This disparity may be related to the region’s varying levels of effective and efficient use of water supplies, sanitation initiatives, and deworming programs. In metropolitan places such as Debre Tabor, the low frequency of soil‐transmitted helminths might be a result of better cleanliness or the effects of mass drug administration initiatives. Nonetheless, the significant frequency of protozoan infections points to ongoing problems with the study area’s water quality and sanitation.

In our study, IPIs were considerably greater in patients with advanced WHO clinical stages [3, 4] (28.3%) than in those in Stages 1‐2 (3.2%). According to a study conducted in Gondar, Ethiopia, patients were more susceptible to IPIs in the latter phases [28]. The natural course of HIV is shown in this pattern, wherein several pathogens, including IPIs, are significantly associated to growing immunosuppression. On the other hand, those who had been on ART for more than 10 years showed a significantly lower IPI (4.8%), suggesting that the immune recovery benefits of long‐term ART treatment may protect against IPIs. The fact that *G. lamblia* and *E. histolytica* are seen in advanced stages emphasizes how important these infections are in defining AIDS. Evidence from Nekemte, Ethiopia, which shows that long‐term ART decreased IPI risk because of immunological recovery, supports this [29]. This highlights how ART helps to restore the immune system and lessens vulnerability to IPIs. Even long‐term ART users, however, were not completely risk‐free, highlighting the necessity of continuous screening and preventative actions.

### 5.1. Limitation of the Study

The present study provided a comprehensive data on the frequency of IPIs and identified important factors linked with it by targeting those high‐risk groups (individuals with HIV/AIDS). However, the use of convenience sampling and considering a single study site may be considered as key limitations in our study as it might affect the generalizability of our findings to the broader population.

## 6. Conclusions and Recommendations

This study confirms that among HIV patients receiving ART at DTCSH, the frequency of IPIs was notably high (33.5%), and that there is a significant correlation between low CD_4_ counts, advanced WHO stage, and shorter ART duration. The necessity of screening and treating the two types of parasite coinfections was highlighted by these data, which showed that the burden of these infections persisted even after ART became available. All HIV patients on ART must have IP testing, with those with low CD_4_ levels and advanced disease stages (WHO 3‐4) receiving priority. Furthermore, our results showed that rural residents had much higher IPIs. This demonstrated the necessity of guaranteeing consistent access to potable water and adequate sanitation. Additionally, mass drug administration programs should be increased, with a focus on the rural population, and patients and caregivers should be taught about basic cleanliness, such as handwashing and food safety procedures. Furthermore, it is advised to conduct extensive prospective studies to examine the long‐term effects of ART on parasite infections.

**Nomenclature**
AIDS: Acquired Immune Deficiency Syndrome
ART: Antiretroviral therapy
CD_4_
: Cluster of differentiation
DTCSH: Debre Tabor Comprehensive Specialized Hospital
IP: Intestinal parasite
HIV: Human immunodefficiency virus
SOP: Standard operating procedure
SPSS: Statistical Package for Social Science
WHO: World Health Organization

## Author Contributions

Dessie Tegegne, Getu Abeje, Mulu Kebede, and Yikeber Abateneh work on the conceptualization, methodology, data curation, formal analysis, writing the original manuscript, and interpretation of the data. Getaneh Tegegne, Teklehaimanot Kiros, Mulat Erkihun, Andargachew Almaw, Ayenew Assefa, Ayenew Berhan, Alemie Fentie, Tegenaw Tiruneh, Birhanemaskal Malkamu, Mahider Shimelis Feyisa, Biruk Legese, Sisay Getu, Mitikie Wondimagegn Meberat, Birhanu Getie, Shewaneh Damtie, and Bekele Sharew all work on the project administration, supervision of all the activities, interpretation of the data, and play a role in formal analysis.

## Funding

The authors have nothing to report.

## Disclosure

All authors have read and approved the final version of the manuscript for publication and give the permission to publish this manuscript in this journal. All the authors are responsible and accountable for the accuracy and originality of the data.

## Ethics Statement

Ethical clearance was obtained from the Research and Community Service Review Committee of the College of Health Sciences at Debre Tabor University with a reference number RCS/12//666/2025. Prior to data collection, authorization to access medical records was obtained from the DTCSH. All personal identities were anonymized throughout data extraction and analysis in order to carefully safeguard patient confidentiality. Because the study used retrospective data, there was no need for direct patient interaction.

## Consent

The authors have nothing to report.

## Conflicts of Interest

The authors declare no conflicts of interest.

## Data Availability

The data that support the findings of this study are available from the corresponding author upon reasonable request.
